# Exploration of Lipid Metabolism Alterations in Children with Active Tuberculosis Using UHPLC-MS/MS

**DOI:** 10.1155/2023/8111355

**Published:** 2023-02-09

**Authors:** Baixu Sun, Fang Liu, Qingqin Yin, Tingting Jiang, Min Fang, Li Duan, Shuting Quan, Xue Tian, Adong Shen, Kaixia Mi, Lin Sun

**Affiliations:** ^1^Beijing Children's Hospital, Capital Medical University, National Clinical Research Center for Respiratory Diseases, National Key Discipline of Pediatrics, Capital Medical University, Key Laboratory of Major Diseases in Children, Ministry of Education, Beijing Pediatric Research Institute, National Center for Children's Health, Beijing, China; ^2^Baoding Children's Hospital, Baoding, Hebei, China; ^3^Department of Pediatrics Infectious Diseases, The No. 1 People's Hospital of Liangshan Yizu Autonomous Prefecture, Liangshan, China; ^4^CAS Key Laboratory of Pathogenic Microbiology and Immunology, Institute of Microbiology, Chinese Academy of Sciences, Beijing, China

## Abstract

Metabolic profiling using nonsputum samples has demonstrated excellent performance in diagnosing infectious diseases. But little is known about the lipid metabolism alternation in children with tuberculosis (TB). Therefore, the study was performed to explore lipid metabolic changes caused by *Mycobacterium tuberculosis* infection and identify specific lipids as diagnostic biomarkers in children with TB using UHPLC-MS/MS. Plasma samples obtained from 70 active TB children, 21 non-TB infectious disease children, and 21 healthy controls were analyzed by a partial least-squares discriminant analysis model in the training set, and 12 metabolites were identified that can separate children with TB from non-TB controls. In the independent testing cohort with 49 subjects, three of the markers, PC (15:0/17:1), PC (17:1/18:2), and PE (18:1/20:3), presented with high diagnostic values. The areas under the curve of the three metabolites were 0.904, 0.833, and 0.895, respectively. The levels of the altered lipid metabolites were found to be associated with the severity of the TB disease. Taken together, plasma lipid metabolites are potentially useful for diagnosis of active TB in children and would provide insights into the pathogenesis of the disease.

## 1. Introduction

As one of the oldest and most formidable human pathogens, *Mycobacterium tuberculosis* (MTB) infection remains responsible for the largest number of deaths worldwide from a single infectious disease. Tuberculosis (TB) in children is increasingly recognized as making up a considerable part of the global TB burden, accounting for 11% of these and 16% of TB-associated deaths [1]. Because of their immature immune systems, children usually present with more rapid and severe disease progress after MTB infection. However, the management of TB in children is difficult because of limitations in the current diagnostic methods and the paucibacillary nature of TB disease in children [2]. For example, microbiological culture is not appropriate for rapid detection because of the low sensitivity and time-consuming characteristic [3]. Molecular diagnostic tools using respiratory samples are also limited by the difficult acquisition of samples with high quality [4]. So, respiratory specimen-independent diagnostic methods are urgently needed nowadays.

It is hoped that more sensitive high-throughput technologies may provide new insights into the early diagnosis of TB without existing microbiological evidence [5]. The understanding of the metabolic profiling of the subjects who are infected with MTB may aid in our understanding of the mechanisms of TB and develop new diagnostic methods. The metabolic profiling has demonstrated excellent performance in the discrimination of TB patients from healthy controls [6, 7]. Lipidomics has been extensively used to research specific plasma metabolites and key pathways which related to many diseases [8, 9]. Previous lipidomic studies have identified potential plasma biomarkers associated with COVID-19 infection [10], Ebola virus disease [11], gestational diabetes [12], IgG4-related diseases [13], and cardiovascular diseases [14]. Children are considered to have different responses to infection compared with adults because of the immature immune system [15, 16]; however, there are few studies investigating specific plasma lipid metabolic biomarkers of children.

As an intracellular parasitic bacterium, MTB survives within mononuclear cells, where it successfully combats macrophage microbicidal mechanisms through host–pathogen interactions, such as by regulating the lipid metabolism of the host [17, 18]. At the initial stage of infection, MTB can induce the accumulation of cholesteryl ester and glyceride, leading to the formation of foamy macrophages and tuberculous granuloma [19]. Once MTB was released from pulmonary granuloma, the failure to limit the infection can exacerbate disease symptoms and further contribute to severe disease types. MTB has evolved a wide array of specific lipids and related metabolisms that actively interact with the immune response and lipid metabolism of the host [20]. As results of the interactions, MTB can induce the anti-TB responses or cause tissue injury of the host because of excessive inflammation [21, 22]. This process causes corresponding symptoms and signs. Therefore, the lipid metabolic spectrum of the host is implicated in the pathogenesis of MTB infection.

Metabolome analyses using ultra-high-performance liquid chromatography coupled with mass spectrometry (UHPLC-MS/MS) have advantages because of its high sensitivity and selectivity and good time-retention reproducibility [23, 24]. In the present study, UHPLC-MS/MS analysis was used to identify differential lipid metabolites in children with active TB compared with healthy controls (HC) or diseased controls (DC). Metabolite profiles associated with TB disease severity were also analyzed. The potential biomarkers showed good diagnostic accuracy for distinguishing active TB from HC and DC and contributed to the pathogenesis of TB.

## 2. Materials and Methods

### 2.1. Study Participants

Subjects were recruited between February 10, 2020, and June 30, 2021, at the No. 1 People's Hospital of Liangshan Yizu Autonomous Prefecture and Baoding Children's Hospital. In accordance with the Chinese and World Health Organization guidelines, patients were enrolled with suspected TB if they had the following symptoms: a cough lasting for more than 2 weeks, weight loss, malnutrition, tuberculosis contact, and/or a positive chest radiograph. The patients were then diagnosed with either active TB or non-TB infectious diseases. A diagnosis of active TB was based on the following factors: (1) positive MTB culture, (2) at least one TB symptom or sign, (3) radiographic evidence consistent with TB, (4) positive tuberculin skin test (TST) or IGRA, and (5) clinical and radiological improvement following anti-TB chemotherapy. Tuberculous meningitis, miliary TB, and disseminated TB were defined as severe forms of TB.

Children with non-TB infectious diseases were enrolled in the DC group if they were symptomatic but did not fit the TB diagnostic criteria and had confirmed etiological evidence of infection with a virus, mycoplasma, or bacteria other than MTB. In the HC group, healthy children were enrolled from those who were admitted for physical examination. Children with latent tuberculosis infection were excluded from the DC and HC groups.

In total, 70 active TB children, 21 disease controls, and 21 healthy controls were recruited to serve as the training set. The independent validation cohort enrolled 30 children from the active TB group, 10 from the disease group, and 9 from the healthy group.

This retrospective study was approved by the Ethics Committees of Beijing Children's Hospital (No. 2022-E-136-Y). Written informed consent was obtained from the guardians of all patients.

### 2.2. Sample Preparation and Lipid Extraction

Sample preparation and lipid extraction were performed according to the MTBE protocol [25]. Whole blood was collected in EDTA tubes and centrifuged at 3,000 × g for 10 min at 4°C within 4 h of collection. Next, 0.75 mL of methanol and 2.5 mL of methyl tertiary butyl ether (MTBE) were successively added to 100 *μ*L of plasma, and the mixture was incubated for 1 h at room temperature on a shaker. Phase separation was induced by adding 0.625 mL of MS-grade (mass spectrometry grade) water. After 10 min, the sample was centrifuged at 1,000 × g for 10 min to collect the upper phase. The lower phase was reextracted with 1 mL of the solvent mixture (MTBE/methanol/water [10 : 3 : 2.5, *v*/*v*/*v*]), and the upper phase was collected. The combined organic phases were dried and dissolved in 100 *μ*L of isopropanol for storage before analysis using UHPLC-MS/MS. The blank extracted sample was used to control the background impurities. It was a matrix of the experimental sample, and the pretreatment process was the same as the experimental sample.

### 2.3. UHPLC-MS/MS Analysis

UHPLC-MS/MS analyses were performed using a Vanquish UHPLC system (Thermo Fisher, USA) coupled with an Orbitrap Q Exactive^TM^ HF mass spectrometer (Thermo Fisher) at Novogene Co., Ltd. (Beijing, China). Samples were injected into a Thermo Accucore C30 column (150 × 2.1 mm, 2.6 *μ*m) using a 20 min linear gradient at a flow rate of 0.35 mL/min. The column temperature was set at 40°C. Elution gradient was performed using a binary solvent system consisting of acetonitrile/water (6/4) with 10 mM ammonium acetate and 0.1% formic acid (solvent A) and acetonitrile/isopropanol (1/9) with 10 mM ammonium acetate and 0.1% formic acid (solvent B). The solvent gradient was set as follows: 30% B, initial; 30% B, 2 min; 43% B, 5 min; 55% B, 5.1 min; 70% B, 11 min; 99% B, 16 min; 30% B, 18.1 min. The Q Exactive^TM^ HF mass spectrometer was operated in positive [negative] polarity mode with sheath gas: 20 arbitrary units; sweep gas: 1 arbitrary unit; auxiliary gas rate: 5; spray voltage: 3 kV; capillary temperature: 350°C; heater temperature: 400°C; S-Lens RF level: 50; scan range: 114–1700 *m*/*z*; automatic gain control target: 1*e*6; normalized collision energy: 25 eV, 30 eV [20 eV, 24 eV, 28 eV]; injection time: 100 ms; isolation window: 1 *m*/*z*; automatic gain control target (MS2): 1*e*5; dynamic exclusion: 15 s. Mass spectra were acquired using data-dependent acquisition in both positive and negative polarity. Polarity switch was used for electrospray ionization. Quality control (QC) samples were used to access recovery and instrumental variations as previous studies [26, 27]. After lipid extraction, equal volumes of each plasma were pooled to create a QC sample. The QC sample was reinjected into each batch to monitor and correct analytical bias. Lipids are easily disturbed by external factors and change rapidly. We will ensure the quality of the final collected data through the correlation quality control of QC samples.

### 2.4. Data Search

The raw data files generated by UHPLC-MS/MS were processed using Compound Discoverer 3.01 (CD3.1, Thermo Fisher) to perform peak alignment and peak picking for each metabolite. The parameters for data processing were set as follows: retention time tolerance, 0.2 min; actual mass tolerance, 5 ppm; signal intensity tolerance, 30%; signal/noise ratio, 3; and minimum intensity, 100,000. After the initial processing, peak intensities were normalized to the total spectral intensity. Normalization was performed by correcting the area of the MS picks across the batches using the QC pooled samples and by centering their values around the mean of the QC areas. The normalized data were then used to predict the molecular formula based on additive ions, molecular ion peaks, and fragment ions. Peaks were then matched with the Lipidmaps (http://www.Lipidmaps.org/) and Lipidblast databases (https://fiehnlab.ucdavis.edu/) to obtain accurate qualitative and relative quantitative results.

### 2.5. Data Analysis

Firstly, the principal component analysis (PCA) was performed to evaluate the data quality of lipid metabolites in terms of homogeneity and reproducibility. And then, the partial least-squares discriminant analysis (PLS-DA) method was applied to explore metabolic differences between groups. Metabolites with variable importance in the projection (VIP) > 1, *P* value < 0.05, and fold change (FC) ≥ 1.5 or ≤ 0.67 were considered differential metabolites. The differential metabolites were then subjected to pathway analysis using Metabo Analyst (https://www.metaboanalyst.ca), followed by visualization.

### 2.6. Statistical Analysis

Statistical analysis of the differential metabolites and clinical data was performed using SPSS 22.0, GraphPad Prism 8.0, and Metabo Analyst 5.0. Values are expressed as frequencies, percentages, means ± standard deviations (SDs), or medians (Q25, Q75). Continuous data were analyzed using *t*-tests, and categorical data were analyzed using Fisher's exact test. Significant correlations were calculated using the Pearson correlation test. Receiver-operating characteristic (ROC) curve analysis was used to evaluate the diagnostic performance of potential biomarkers. The significance level was set at *P* < 0.05 for all tests.

## 3. Results

### 3.1. Clinical Characteristics

In this study, 100 children with active TB, 31 children with infectious diseases other than TB, and 30 healthy children were enrolled. The children aged from 0.3 to 14 years, and 91 (56.5%) cases were male. Among the children with active TB, 68 children (68.0%) had positive MTB culture results or molecular testing results using Xpert MTB/RIF Ultra. Furthermore, 45 children (45.0%) had severe types of TB (29 had tuberculous meningitis, eight had miliary TB, and eight had disseminated TB). All children in the DC and HC groups were IGRA negative. The demographic characteristics of the subjects in training and validation set are shown in Table 1.

### 3.2. Differential Lipid Metabolites between Active TB Children and Non-TB Children

**Figure 1:**
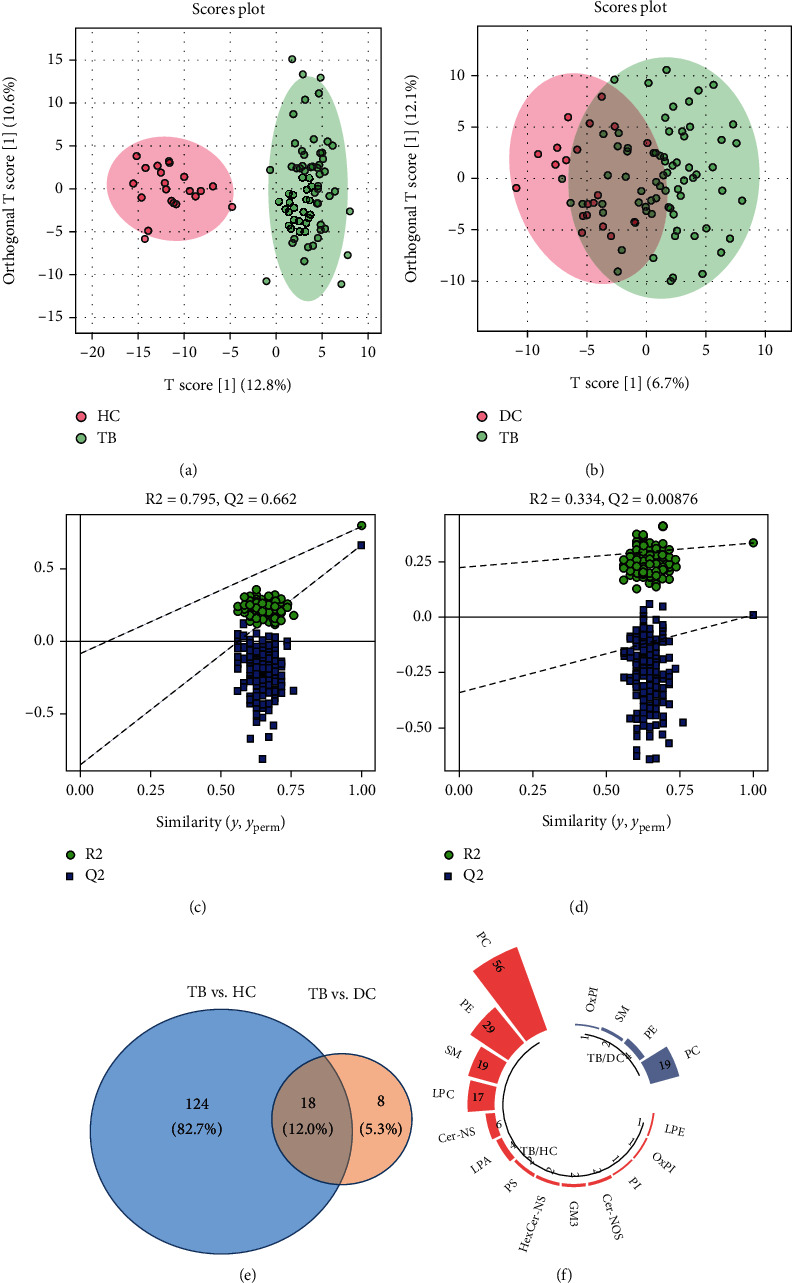
Identification of differential lipid metabolites in children with active TB. (a) PLS-DA score plots for the children with TB and HC; (b) PLS-DA score plots for the children with TB and DC; (c) PLS-DA parameters for the children with TB and HC; (d) PLS-DA parameters for the children with TB and DC; (e) differentially expressed lipid metabolites identified in TB children compared with DC and HC; (f) major classes of plasma differential lipid metabolites.

PCA was applied to visualize the distributions of the control groups, TB group, and QC samples. All QC samples were tightly clustered together in the center of the PCA score plot, reflecting the stability of the instrument and showing that the quality of all the LC–MS data for this study was reliable and acceptable (Figure S1). After baseline filtering, peak recognition, peak alignment, and normalization, 351 lipid metabolites were identified in all the enrolled samples. To visualize the metabolic differences specific to active TB and evaluate the data quality of the metabolic profiles, a PLS-DA model was used (see Figures 1(a) and 1(b)). The parameters of the PLS-DA model are shown in Figures 1(c) and 1(d). Lipid metabolites that were differential between TB patients and HC or DC were structurally identified; there were 142 differential lipid metabolites between TB patients and HC and 26 between TB patients and DC. Variables with VIP > 1, *P* < 0.05, and FC ≥ 1.5 or ≤ 0.67 were considered potential markers. Finally, 18 overlapping metabolites were selected (see Figure 1(e)). The major classes of plasma differential lipid metabolites were phosphatidylcholine (PC) and phosphatidylethanolamine (PE) (see Figure 1(f)).

### 3.3. Potential Biomarkers for Active TB Diagnosis

**Figure 2:**
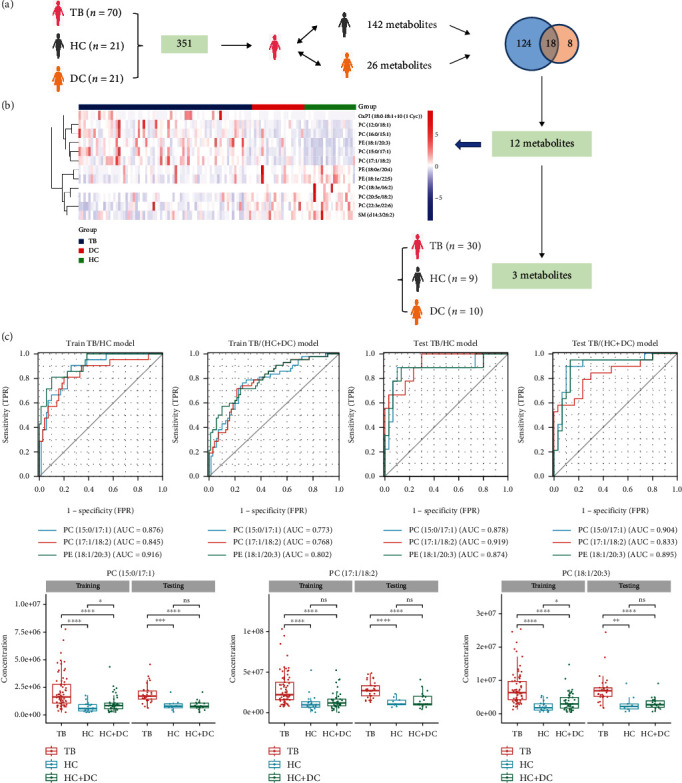
Verification and evaluation of potential lipid biomarkers for the classification of TB patients. (a) The workflow for biomarker selection; (b) the heatmap of 12 differential lipid metabolites in TB, DC, and HC groups; (c) AUC values of three biomarkers for the classification of TB and non-TB.

Based on the screening of differential metabolites between TB group and HC or DC group (see Figure 2(a)), the potential discriminant biomarkers were evaluated by ROC analysis to assess their sensitivity and specificity. Among 18 overlapping lipid metabolites, 12 of them showed high diagnostic values for discrimination of TB versus non-TB children. All 12 lipid metabolites are shown in Table 2; six lipids were increased, and six lipids were decreased in TB patients. A heatmap was generated to provide an intuitive visualization of the content variation of the differential lipids (see Figure 2(b)). The lipid metabolites were able to be used to distinguish TB patients from the control groups, which indicates that they are associated with the pathogenesis of active TB in children.

To further verify these 12 lipids as potential diagnostic biomarkers, a separate and blinded set was used for validation. We found that three lipid metabolites have good diagnostic performance in distinguishing TB patients from non-TB children (see Figure 2(c)). All the three lipid metabolites were markedly increased in children with active TB. For discrimination of TB versus non-TB children (HC and DC groups) in the training cohort, the area under the ROC curves (AUC) of PC (15:0/17:1), PC (17:1/18:2), and PE (18:1/20:3) were 0.773, 0.768, and 0.802, respectively. The AUCs were 0.904, 0.833, and 0.895, respectively, in the validation cohort. In order to ensure the reliability of this result, we used the same method to randomly generate 5 groups of training sets and test sets, and the corresponding results were consistent with the above (see Figure S2). The detailed diagnostic index of these three lipid metabolites is shown in Table 3.

### 3.4. Association of Lipid Metabolites with Clinical Phenotypes

**Figure 3:**
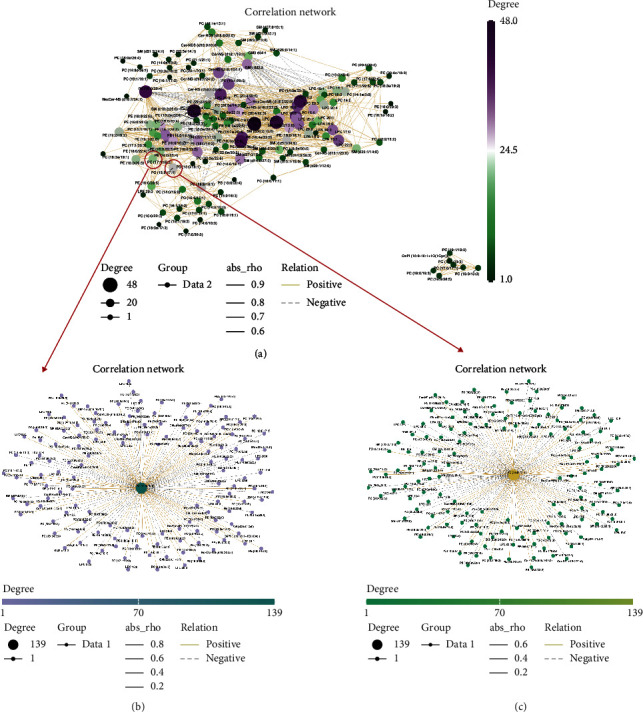
Differential correlation analyses of plasma lipids in TB patients relative to HC. (a) Correlation analyses of all the identified lipid metabolites between TB and HC; (b) correlation analyses of PC (15:0/17:1); (c) correlation analyses of PC (17:1/18:2).

To decipher the relationship between metabolites, we then looked for pathologically relevant lipid modules in TB children relative to healthy controls, using Cytoscape to construct networks from differentially correlated lipid pairs. Only differential correlations with empirical *P* < 0.05 were displayed (see Figure 3(a)). The modules of PC (15:0/17:1) and PC (17:1/18:2) in the global network were circled and enlarged (see Figures 3(b) and 3(c)). The correlations between the levels of these two metabolites implied that these metabolites lie along a common metabolic pathway and are coregulated. Changing correlation patterns between lipid-pairs in TB compared to healthy states potentially indicated pathologically relevant metabolic dysregulation.

**Figure 4:**
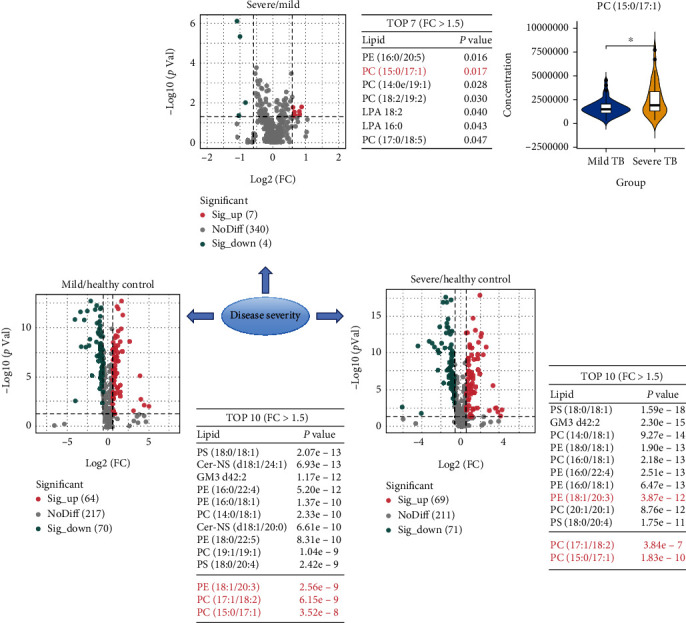
Lipid changes in TB patients with increasing severity.

The lipids PC (15:0/17:1), PC (17:1/18:2), and PE (18:1/20:3) were significantly increased in children with severe or mild TB compared with healthy controls. The concentration of PC (15:0/17:1) showed significantly increased trend in severe TB compared with mild TB children (see Figure 4). Children with severe active TB were more likely to present with a weaker immune response, higher bacterial loads, and more severe symptoms (see Table S1). The level of plasma PC (15:0/17:1) was associated with the clinical phenotypes of severe active TB in children (see Figure S3).

## 4. Discussion

In recent years, unexpected advances have been achieved in the diagnosis of active TB; the early and accurate identification of TB in children remains dismal due to the lack of efficient diagnostic tests. Therefore, exploring novel biomarkers will open a new window to the clinical diagnosis of childhood TB. As the ultimate downstream pool of genome transcription, the metabolites can reflect changes in the biochemistry of living cells or organisms more directly, when compared with genetics and proteomics [28]. Therefore, the metabolites underlying the dysregulated metabolic pathways can be defined as biomarkers to diagnose the disease and reflect the disease progression.

To verify this hypothesis, we employed the sensitive UHPLC-MS/MS method to analyze plasma metabolomes in both children with active TB and non-TB controls. Finally, we discovered three lipid metabolites, PC (15:0/17:1), PC (17:1/18:2), and PE (18:1/20:3), which presented good value in diagnosis of active TB. Host plasma is rich in lipids, which are the major nutrition source for the growth and reproduction of MTB. In addition to regulating the immune cells of the host, infection with MTB can also regulate lipid metabolism. Our results were consistent with those of a metabolomic analysis of patients with osteoarticular TB, in which PC and PE were upregulated [29].

The strong correlation between lipid metabolites and the pathophysiological factors of the disease made these lipids the ideal biomarkers of TB in children. They have potential value for clinical application and may encourage early diagnosis and guide the successful development of novel therapeutic strategies. Although many attempts have been made to combine metabolites into a diagnostic signature for TB in adults [30, 31], there have been comparatively few pediatric studies. A study in India reported that N-acetylneuraminate is a diagnostic biomarker for active TB in children, with an AUC of 0.66 [32]. In our study, the single lipid metabolites PC (15:0/17:1), PC (17:1/18:2), and PE (18:1/20:3) had an AUC > 0.8, thus representing better sensitivity than MTB culture and a similar sensitivity to Xpert MTB/RIF Ultra in this population. These nonsputum diagnostic biomarkers in symptomatic children deliver promising results. Among the biomolecules that compose total cell biomass, lipids have received relatively little research attention. PC is a typical eukaryotic membrane phospholipid that is fundamental for symbiotic and pathogenic microbe–host interactions [33, 34]. There is evidence that PC as well as the choline metabolites derived from its synthesis and catabolism contributes to both proliferative growth and programmed cell death [35]. PE makes up the backbone of most biological membranes. In healthy cells, PE resides predominantly in the inner leaflet of the cell membrane, while in dead or dying cells, PE is externalized to the outer leaflet of the plasma membrane. Therefore, new molecular technology for the molecular imaging of cell death based on PE exposure on the cell surface may have future potential for disease diagnosis [36].

Metabolomics refers to the quantitative measurement of dynamic metabolic changes associated with specific clinical phenotypes [37]. Therefore, in the context of investigating a disease, such changes may be useful for better disease characterization, improved diagnostics and treatment, and other clinical applications. Various sample types, including blood (serum and plasma), sputum, urine, tissue, and bacteriological cultures, have been used to identify new metabolomic biomarkers. Because of the difficulty of obtaining respiratory tract specimens from children and the relatively low bacterial load, sputum is not ideal for metabolic testing. By contrast, plasma has several advantages, including the ability to extract systemic metabolomic changes caused by an infection and to investigate the metabolic changes of pulmonary and extrapulmonary TB [7]. It also reflects the disease phenotype because it reveals systemic alterations in the host caused by both infection and treatment [7].

In the present study, we also identified a differential lipid metabolic signature between children with severe TB and those with mild TB. A relative abundance of target lipids was associated with the progress of patients with severe TB. Many previous studies have investigated why some individuals develop severe TB while others do not. It is generally considered that MTB has evolved to fine-tune the immune response, ultimately modulating the pathogenesis of TB [38]. Studies have revealed that one mechanism of protection against or susceptibility to MTB in a host is the ability of specific lipids to either directly or indirectly regulate cell death outcomes of infected macrophages, which play pivotal roles in TB pathogenesis [39, 40]. In our study, an increased abundance of PC (15:0/17:1) was associated with an increased bacterial load of MTB in vivo as well as a severe clinical phenotype and symptoms. Our results were consistent with those of a previous study in which the balance between lipid metabolites and the immune response was associated with the control of bacillary growth and lung pathology [41].

In conclusion, we used UHPLC-MS/MS technology to explore the lipid metabolism alterations in children with active TB. Three significantly increased lipids—PC (15:0/17:1), PC (17:1/18:2), and PE (18:1/20:3)—were able to distinguish children with active TB from healthy children and those with other infectious diseases. The levels of the altered lipid metabolites were found to be associated with the severity of the TB disease. Our study also provides a new perspective for developing novel diagnostic methods and understanding the pathogenesis of TB.

## Figures and Tables

**Table 1:** Demographic characteristics of the participants.

Characteristics	Training set (*n* = 112)			Independent testing set (*n* = 49)		
	TB	HC	DC	TB	HC	DC
Sample size	70	21	21	30	9	10
Gender (male/female)	37/33	13/8	14/7	15/15	6/3	6/4
Age (years)^a^	8.9 (6.8-12.0)	8.0 (3.9-11.2)	6.4 (3.5-9.0)	8.9 (6.0-12.0)	9.2 (7.4-11.1)	3.7 (2.5-4.0)
Age range (years)	0.3-14.0	1.0-11.0	0.6-13.2	0.8-13.0	0.7-11.0	5.8-13.0
Location of TB (pulmonary/extrapulmonary)	33/37	/	/	15/15	/	/
Severity of TB (severe/nonsevere)	35/35	/	/	10/20	/	/

TB: tuberculosis; HC: healthy control; DC: disease control. ^a^Data are presented as mean (interquartile range).

**Table 2:** Details of the differential lipid metabolites between the active TB and the non-TB groups.

Metabolites	Polarity for quantitation	Formula	Molecular weight	RT (min)	TB vs. HC				TB vs. DC			
					FC	*P* value	VIP	Trend	FC	*P* value	VIP	Trend
OxPI (18:0-18:1+1O(1Cyc))	Negative	C45 H83 O14 P	878.5533	8.99	10.40	2.20*e*-05	1.67	↑	6.21	4.24*e*-02	1.46	↑
PC (12:0/18:1)	Positive	C38 H74 N O8 P	703.5152	9.46	2.49	1.02*e*-04	1.87	↑	1.65	3.96*e*-02	1.36	↑
PC (16:0/15:1)	Positive	C39 H76 N O8 P	717.5311	10.00	3.97	5.28*e*-07	1.16	↑	1.57	4.14*e*-02	1.32	↑
PE (18:1/20:3)	Positive	C43 H78 N O8 P	767.5468	13.16	3.47	8.40*e*-09	1.33	↑	1.53	9.46*e*-03	1.59	↑
PC (15:0/17:1)	Positive	C40 H78 N O8 P	731.5467	12.57	3.08	1.95*e*-07	1.21	↑	1.55	1.88*e*-02	1.51	↑
PC (17:1/18:2)	Positive	C43 H80 N O8 P	769.5641	12.56	2.44	1.83*e*-06	1.10	↑	1.59	8.24*e*-03	1.70	↑
PE (18:0e/20:4)	Negative	C43 H80 N O7 P	753.5683	13.18	0.47	1.02*e*-05	1.18	↓	0.55	1.05*e*-02	1.45	↓
PE (18:1e/22:5)	Negative	C45 H80 N O7 P	777.5684	13.37	0.55	3.34*e*-06	1.36	↓	0.66	6.21*e*-03	1.84	↓
PC (18:3e/16:2)	Positive	C42 H76 N O7 P	737.5371	9.56	0.09	1.05*e*-08	1.10	↓	0.59	1.27*e*-02	1.64	↓
PC (20:5e/18:2)	Positive	C46 H80 N O7 P	789.5663	9.79	0.53	3.51*e*-04	1.11	↓	0.61	2.18*e*-04	2.72	↓
PC (22:3e/22:6)	Positive	C52 H88 N O7 P	869.6272	12.19	0.57	1.32*e*-05	1.09	↓	0.65	1.85*e*-03	1.88	↓
SM (d14:3/26:2)	Positive	C45 H83 N2 O6 P	778.5984	10.16	0.43	8.17*e*-07	1.43	↓	0.61	2.87*e*-03	2.08	↓

TB: tuberculosis; HC: healthy control; DC: disease control; FC: fold change; VIP: variable importance in the projection.

**Table 3:** The differential lipid metabolites for the diagnosis of active TB.

Metabolites	AUC	Sensitivity (%)	Specificity (%)	Youden Index	Best cut-off value
PC (15:0/17:1)	0.904	90.0	89.5	0.795	1.13*e*+06
PC (17:1/18:2)	0.833	76.7	78.9	0.556	2.07*e*+07
PE (18:1/20:3)	0.895	86.7	94.7	0.814	5.02*e*+06

TB: tuberculosis; AUC: area under the ROC curve.

## Data Availability

All data during this study are included in the published article. Meanwhile, the datasets analyzed during the study are available from the corresponding author upon reasonable request.

## Supplementary Materials

Supplementary 1 Figure S1: PCA visualization analysis of the distribution of TB, HC, and DC groups and QC samples. Figure S2: AUC values of the three candidate biomarkers for the classification of TB and non-TB based on five randomly generated training and test sets. Figure S3: the level of plasma PC (15:0/17:1) in children with different testing results and symptoms.

Supplementary 2 Table S1: clinical characteristics of the participants with severe TB vs. mild TB.
