# Supplementary material for: Exploration of Lipid Metabolism Alterations in Children with Active Tuberculosis Using UHPLC-MS/MS
Source: J Immunol Res. 2023 Feb 9;2023:8111355. doi: 10.1155/2023/8111355 (PMC9936505; doi:10.1155/2023/8111355)
Supplement: Supplementary 1 — Figure S1: PCA visualization analysis of the distribution of TB, HC, and DC groups and QC samples. Figure S2: AUC values of the three candidate biomarkers for the classification of TB and non-TB based on five randomly generated training and test sets. Figure S3: the level of plasma PC (15:0/17:1) in children with different testing results and symptoms. [file 8111355.f1.zip › supplementary figure caption.docx]

Figure S1 PCA visualization analysis of the distribution of TB, HC and DC groups and QC samples.

Figure S2 AUC values of the three candidate biomarkers for the classification of TB and non-TB based on five randomly generated training and test sets.

Figure S3 The level of plasma PC(15:0/17:1) in children with different testing results and symptoms.
