# Supplementary figures and images for: Exploration of Lipid Metabolism Alterations in Children with Active Tuberculosis Using UHPLC-MS/MS
Source: J Immunol Res. 2023 Feb 9;2023:8111355. doi: 10.1155/2023/8111355 (PMC9936505; doi:10.1155/2023/8111355)

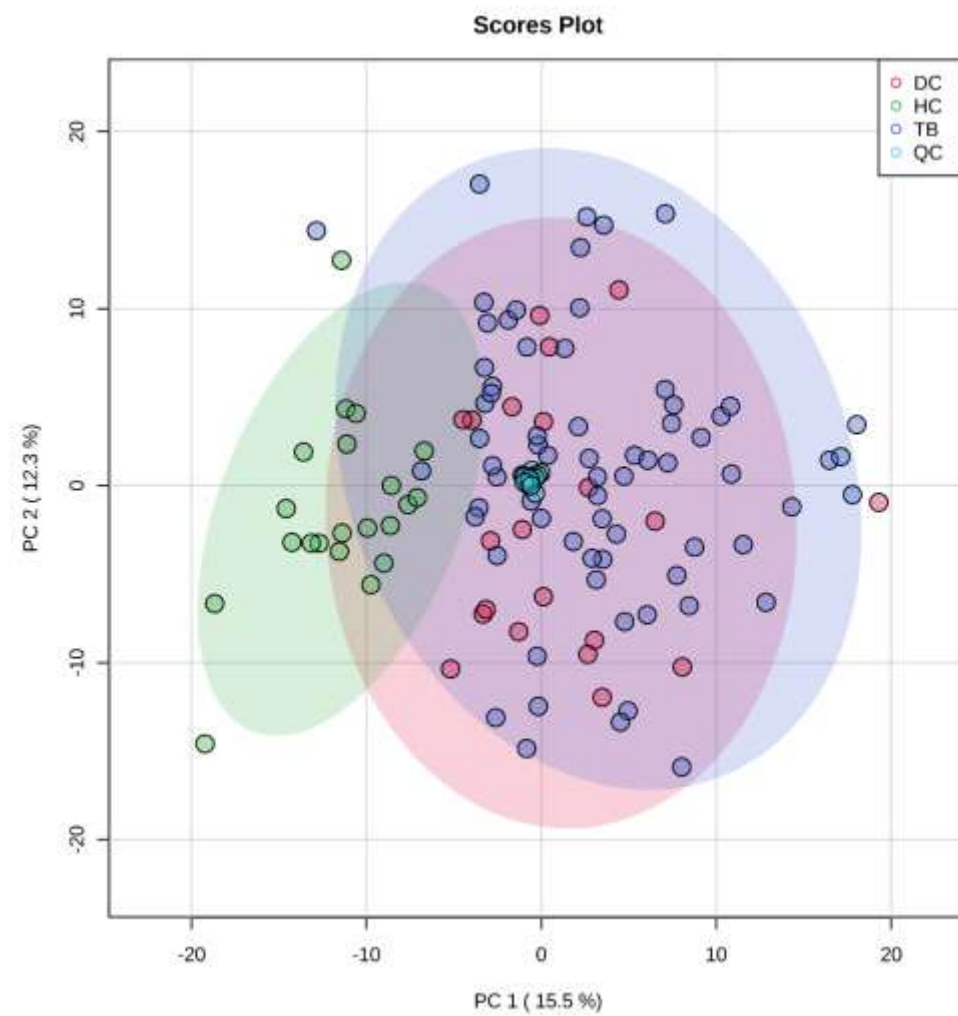

Supplement: Supplementary 1 — Figure S1: PCA visualization analysis of the distribution of TB, HC, and DC groups and QC samples. Figure S2: AUC values of the three candidate biomarkers for the classification of TB and non-TB based on five randomly generated training and test sets. Figure S3: the level of plasma PC (15:0/17:1) in children with different testing results and symptoms. [file 8111355.f1.zip › figure S1 (1).pdf]

a

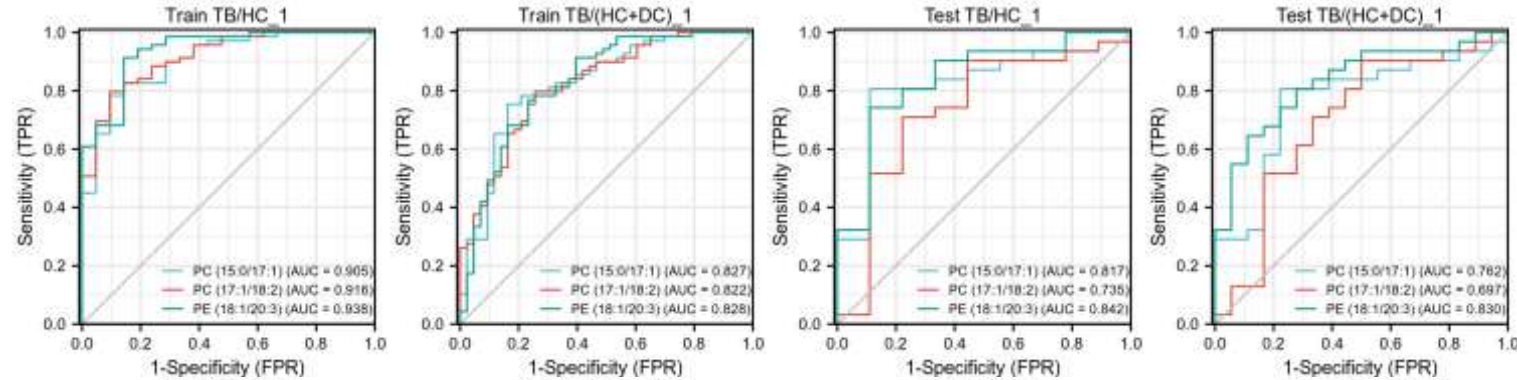

b

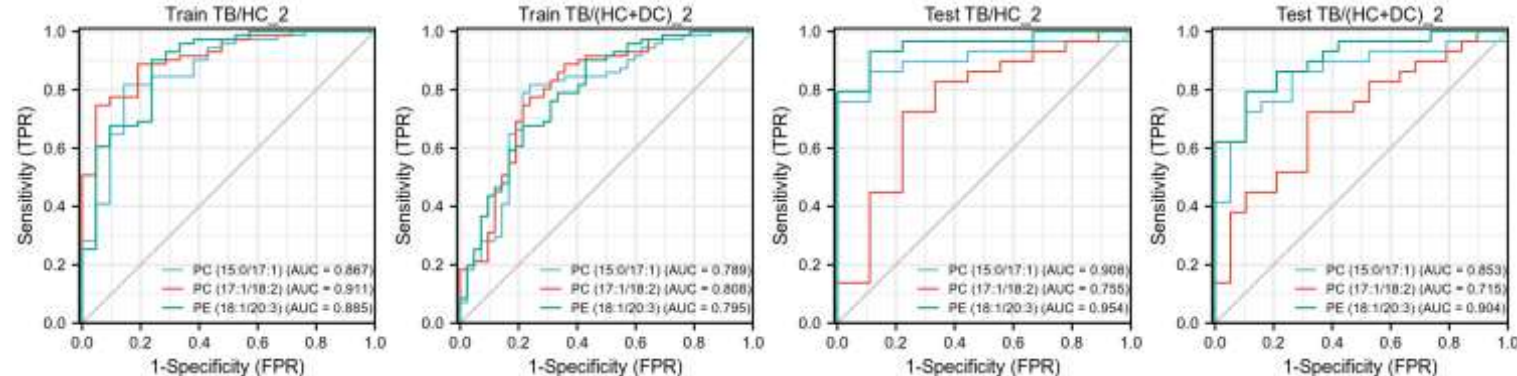

c

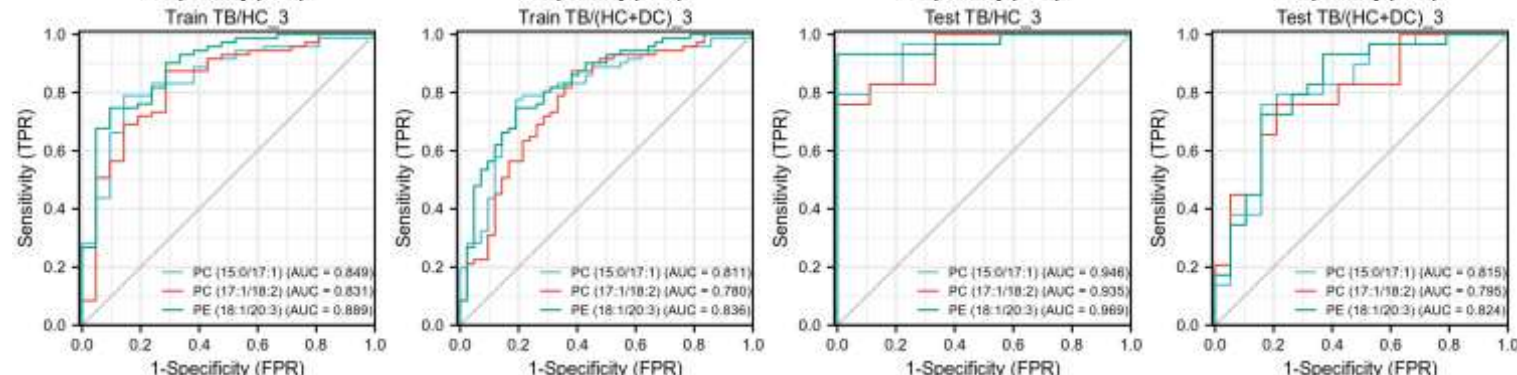

d

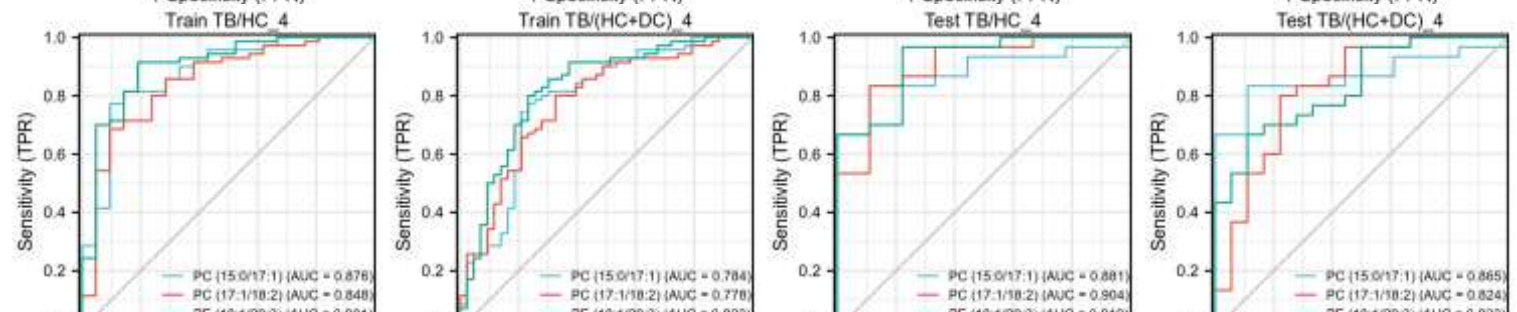

Supplement: Supplementary 1 — Figure S1: PCA visualization analysis of the distribution of TB, HC, and DC groups and QC samples. Figure S2: AUC values of the three candidate biomarkers for the classification of TB and non-TB based on five randomly generated training and test sets. Figure S3: the level of plasma PC (15:0/17:1) in children with different testing results and symptoms. [file 8111355.f1.zip › figure S2 (1).pdf]

a

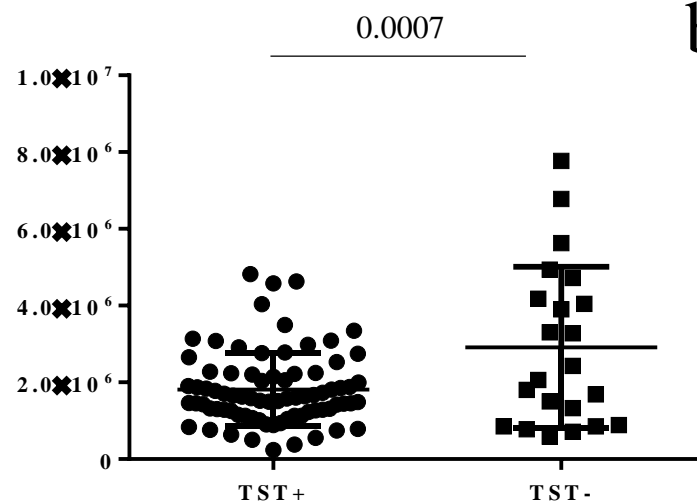

b

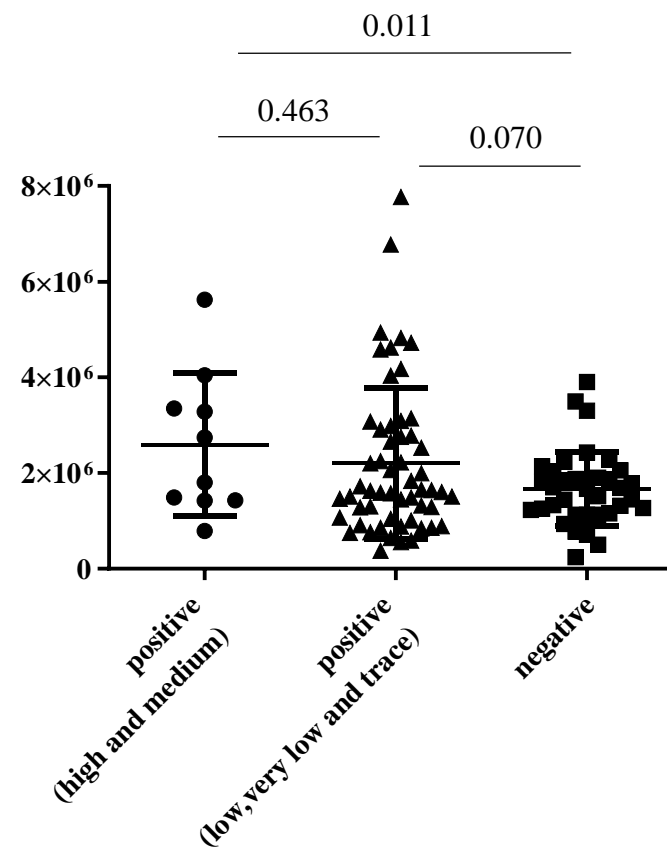

c

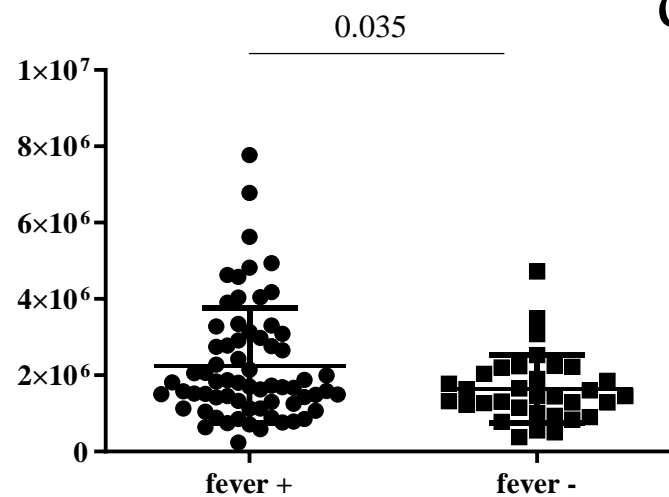

d

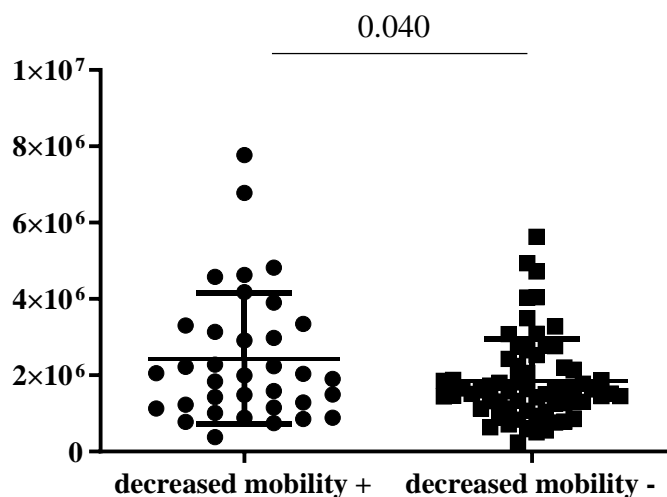

Supplement: Supplementary 1 — Figure S1: PCA visualization analysis of the distribution of TB, HC, and DC groups and QC samples. Figure S2: AUC values of the three candidate biomarkers for the classification of TB and non-TB based on five randomly generated training and test sets. Figure S3: the level of plasma PC (15:0/17:1) in children with different testing results and symptoms. [file 8111355.f1.zip › figure S3 (1).pdf]
