# Supplementary material for: Exploration of Lipid Metabolism Alterations in Children with Active Tuberculosis Using UHPLC-MS/MS
Source: J Immunol Res. 2023 Feb 9;2023:8111355. doi: 10.1155/2023/8111355 (PMC9936505; doi:10.1155/2023/8111355)
Supplement: Supplementary 2 — Table S1: clinical characteristics of the participants with severe TB vs. mild TB. [file 8111355.f2.docx]

Table S1 Clinical characteristics of the participants with severe TB vs. mild TB

| Characteristics | Severe TB (n=45) , n (%) | Non-severe TB (n=55) , n (%) | P value |
| --- | --- | --- | --- |
| **Age (y)** |  |  | 0.307 |
| Mean (SD) | 8.5 (4.1) | 9.3 (3.5) |  |
| **Gender** |  |  | 0.171 |
| Male | 20 (44.4) | 32 (58.2) |  |
| Female | 25 (55.6) | 23 (41.8) |  |
| **TST** |  |  |  |
| Positive | 28 (62.2) | 50 (90.9) | 0.001 |
| Negative | 17 (37.8) | 5 (9.1) |  |
| **IGRAs** |  |  | 0.130 |
| Positive | 37 (82.2) | 51 (92.7) |  |
| Negative | 8 (17.8) | 4 (7.3) |  |
| **Bacterial culture** |  |  | ＜0.001 |
| Positive | 25 (55.6) | 10 (18.2) |  |
| Negative | 20 (44.4) | 45 (81.8) |  |
| **AFB** |  |  | 0.500 |
| Positive | 6 (13.3) | 5 (9.1) |  |
| Negative | 39 (86.7) | 50 (90.9) |  |
| **MTB/RIF Xpert Ultra*** |  |  | ＜0.001 |
| Positive (high and medium) | 7 (15.6) | 3 (5.5) |  |
| Positive (low, very low and trace) | 32 (71.1) | 23 (41.8) |  |
| Negative | 6 (13.3) | 29 (52.7) |  |
| **Symptoms** |  |  |  |
| **Fever** |  |  | ＜0.001 |
| Yes | 40 (88.9) | 29 (52.7) |  |
| No | 5 (11.1) | 26 (47.3) |  |
| **Cough** |  |  | 0.581 |
| Yes | 31 (68.9) | 35 (63.6) |  |
| No | 14 (31.1) | 20 (36.4) |  |
| **Expectoration** |  |  | 0.984 |
| Yes | 23 (51.1) | 28 (50.9) |  |
| No | 22 (48.9) | 27 (49.1) |  |
| **Wheeze** |  |  | 0.974 |
| Yes | 5 (11.1) | 6 (10.9) |  |
| No | 40 (88.9) | 49 (89.1) |  |
| **Anhelation** |  |  | 0.012 |
| Yes | 7 (15.6) | 1 (1.8) |  |
| No | 38 (84.4) | 54 (98.2) |  |
| **Hemoptysis** |  |  | 0.024 |
| Yes | 4 (8.9) | 0 (0) |  |
| No | 41 (91.1) | 55 (100) |  |
| **Chest pain** |  |  | 0.377 |
| Yes | 6 (13.3) | 11 (20.0) |  |
| No | 39 (86.7) | 44 (80.0) |  |
| **Weight does not increase** |  |  | 0.171 |
| Yes | 25 (55.6) | 23 (41.8) |  |
| No | 20 (44.4) | 32 (58.2) |  |
| **Night sweats** |  |  | 0.433 |
| Yes | 24 (53.3) | 25 (45.5) |  |
| No | 21 (46.7) | 30 (54.5) |  |
| **Fatigue** |  |  | 0.028 |
| Yes | 19 (42.2) | 12 (21.8) |  |
| No | 26 (57.8) | 43 (78.2) |  |
| **Anepithymia** |  |  | 0.064 |
| Yes | 28 (62.2) | 24 (43.6) |  |
| No | 17 (37.8) | 31 (56.4) |  |
| **Decreased mobility** |  |  | 0.027 |
| Yes | 21 (46.7) | 14 (25.5) |  |
| No | 24 (53.3) | 41 (74.5) |  |

TB, tuberculosis; TST,tuberculin skin test; IGRA, interferon-γ release assay; AFB, acid-fast bacilli.

^a^ Data are presented as mean (interquartile range).
